# Replicability of Physical Exercise Interventions in Lung Transplant Recipients; A Systematic Review

**DOI:** 10.3389/fphys.2018.00946

**Published:** 2018-07-20

**Authors:** Ruud H. Knols, Nicolas Fischer, Dario Kohlbrenner, Anastasios Manettas, Eling D. de Bruin

**Affiliations:** ^1^Directorate of Research and Education, Physiotherapy & Occupational Therapy Research Center, University Hospital Zurich, Zurich, Switzerland; ^2^Department of Health Sciences and Technology, Institute of Human Movement Sciences and Sport, ETH Zurich, Zurich, Switzerland; ^3^Department of Physiotherapy and Occupational Therapy, University Hospital Zurich, Zurich, Switzerland; ^4^Division of Physiotherapy, Department of Neurobiology, Care Sciences and Society, Karolinska Institute, Stockholm, Sweden

**Keywords:** lung transplant, exercise, tidier checklist, adherence, systematic review, methodological

## Abstract

**Introduction:** This systematic review aimed to assess the replicability of physical exercise interventions in lung transplantation patients. For replicability we focused on (1) the description of training principles, (2) the description of FITT components and adherence to the interventions, (3) the amount of detailed information given on the physical exercise intervention, and (4) reporting the methodological quality of the included works.

**Methods:** Relevant databases (Medline-Ovid, EMBASE, CINAHL, PsychInfo, Cochrane Library) were searched. Author dyads selected and systematically analyzed the included studies independent from each other. A purpose developed checklist was used to assess the details of the exercise interventions and their methodological quality.

**Results:** From the seven included manuscripts, three described resistance training, one endurance, and three combined training approaches. All manuscripts reported specificity and initial values, six manuscripts mention progression and overload. The exercise principle reversibility was reported once and diminishing returns was not reported at all. All studies reported the type of exercise, three studies reported intensity and one study reported time for training. Not any study completely reported frequency or described adherence to the intervention. Lack of detailed reporting was identified as the cause for murky description of the interventions. The highest score for intervention description was 5 of possible 12 items.

**Conclusions:** Replicability of many exercise interventions in LTX is not warranted due too poor descriptions of important items related to training. In particular there were insufficiently detailed reporting of training principles and FITT components in programs developed for LTX. Future interventions that aim to train LTX patients should spent effort in writing reports in which the intervention is detailed to such an extent that full replicability in clinical settings can be guaranteed.

## Introduction

### Background

Survival and health related quality-of-life in patients with end stage pulmonary disease is expected to improve following lung transplantation (LTX) (Hatt et al., 2017). Both in early (less than 1 year) and in late (more than 1 year) LTX patients it seems feasible, safe, and effective to perform physical exercise (PE) following transplantation (Wickerson et al., 2010; Didsbury et al., 2013; Langer, 2015; Wallen et al., 2016). When LTX-exercisers and non-exercisers are compared, some evidence is available supporting that structured PE programs improve maximal exercise capacity, physical function, muscle strength, and bone mineral density (BMD) (Wickerson et al., 2010; Langer et al., 2012) and, thus, favors exercise regarding amelioration of physical and functional task capacities. Such improvements seem especially apparent in patients perceiving their physical functioning as low (Wickerson et al., 2015).

Evidence summaries, however, showed much variability regarding the effects different training programs have on the selected training outcomes for LTX patients. This seems to indicate that not all exercise programs for LTX patients are equally effective (Wickerson et al., 2010; Didsbury et al., 2013; Wallen et al., 2016). These differences in effect might be due to the confusion between exercise and physical activity (Caspersen et al., 1985). Physical activity is “any bodily movement produced by skeletal muscle resulting in energy expenditure” whereas exercise is defined as “a planned, structured, and repetitive subset of physical activity with an identifiable aim to improve or maintain physical fitness” (Caspersen et al., 1985). For clinicians selection and replication of successful exercise programs is important. In addition to evaluating and judging the results of systematic reviews clinicians should, therefore, be able and identify replicable (successful) interventions for the translation of useful interventions in clinical settings (Hoffmann et al., 2014).

Researchers wanting to replicate successful intervention research and clinicians wanting to apply these interventions in their practice both need detailed descriptions of the applied procedures in the intervention program. However, many published intervention research fails to conform to requirements that would guarantee full replicability (Hoffmann et al., 2014). When reporting the results of a PE program, it is important to document the core principles of the used training (Campbell et al., 2012; Winters-Stone et al., 2014) and how these were considered for the population under investigation. These principles of PE are specificity, overload, progression, initial values, reversibility, and diminishing returns; see for a more detailed description (Hoffman, 2002). When designing a PE intervention, adhering to the principles of PE ensures that an appropriate dose and type of exercise can be applied to accomplish a pre-determined training goal; e.g., set goals may relate to endurance, strength and/or physical function. Furthermore, it is of relevance to report the Frequency, Intensity, Time and Type (FITT) of the PE intervention. Only if PE training programs are documented in sufficient detail, their replication can be warranted, and clinicians and researchers are enabled to apply these effective interventions (Hoffmann et al., 2014).

For exercise interventions to be replicable there should at least be reporting of the FITT components (Ammann et al., 2014; Baschung Pfister et al., 2015). This holds true for both more traditional forms of exercise and for innovative training approaches where for example novel technology is used; e.g., exergame based training (Eggenberger et al., 2015; Knols et al., 2016). Randomized controlled trials (RCTs) theoretically provide the best evidence regarding the effectiveness of PE interventions; however, inadequate methodological approaches may overstate treatment effects and bias results (Schulz et al., 2010; Baschung Pfister et al., 2015). Although there are several reviews that describe the outcomes of PE in LTX-patients, to the best of our knowledge there is no overview assessing how well such interventions may be replicated.

### Objectives

This systematic review aimed to assess the replicability of physical exercise programs reported in RCTs investigating the use of PE interventions in LTX patients. To assess replicability we focused on (1) the description of exercise principles, (2) the description of FITT components and adherence to the interventions, (3) the amount of detailed information given on the PE intervention and, (4) reporting the methodological quality of the included works.

## Methods

### Research protocol

This systematic review was performed using a structured study protocol guiding the search strategy, the selection of the studies, and the abridgement of data entry forms for the analysis, similar as in earlier reviews (Ammann et al., 2014; Knols et al., 2016).

### Search strategy and data sources

A librarian experienced in developing searches for systematic reviews (MG; Medical Library of the University of Zurich) developed a purpose-adjusted professional search strategy. The search period covered all years from the start of the medical databases to November 2016, and covered Medline-Ovid, EMBASE, Cochrane Library (PubMed), CINAHL, and PsycInfo. Searches were undertaken using MeSH headings including the following main terms: Organ transplantation, lung transplantation, heart-lung transplantation, lung disease, pulmonary heart disease, thoracic surgery, transplant recipients, chronic obstructive pulmonary disease (COPD), cystic fibrosis, pulmonary hypertension, idiopathic pulmonary fibrosis, lymphangioleiomyomatosis, physical therapy modalities, rehabilitation, exercise movement techniques, exercise therapy, resistance training, physical endurance, physical fitness, motor activity, exercise, perioperative care, balance test, physical activity measures, questionnaire, pedometers or accelerometers, health related quality of life, physical endurance, physical fitness, dyspnea: Borg scale, spirometry, FEV1, peak flow, bone mineral density, range of motion. The following free text words were used: Inspiratory muscle training, exergame or exergaming, robot assisted, high intensity interval training (HIIT), muscle strength, quadriceps and handgrip force, maximal oxygen consumption (VO2max), peak power output (VO2peak), (an-)aerobic threshold, days in hospital, mortality or morbidity, upper extremity endurance capacity, gait speed, sit-to-stand-test, short physical performance battery, timed up and go, 6 min walking test. A detailed description of the search per database is provided in the supplementary file.

Furthermore, from December 2016 to December 2017, the databases were checked monthly by RHK for new publications. The references of all eligible articles and related reviews, as well as recent conference proceedings, were searched and checked through hand searching by NF and RHK. For reporting we used the PRISMA guidelines (Liberati et al., 2009).

### Comparators and interventions

RCTs that contained descriptions of (1) physical exercise training, (2) focused on LTX recipients (including children and adult populations), and (3) described interventions aiming to ameliorate physical function and/or psychosocial outcomes (primary or secondary quantified outcome) were included in the analyses. Studies that included lung transplant candidates that were performing training while being on the LTX waiting list, that included patients with a combined heart and lung transplantation, and were written in non-English languages were excluded.

### Study selection and data extraction

Ordaining manuscript eligibility for inclusion was done by two individuals (NF & RHK) (Kamper et al., 2015) after duplicate removal from the search results. NF & RHK screened all the retrieved citations independent from each other and they, after this, met for a consensus discussion. In this discussion manuscripts where disagreement surrounding eligibility was apparent were reviewed together. In case of remaining disagreement between the reviewers EDB was consulted for a final decision regarding in-/exclusion.

### Data extraction and analysis of the studies

NF created purpose-designed data collection sheets commensurable with approaches used in previous systematic reviews (Ammann et al., 2014; Baschung Pfister et al., 2015; Knols et al., 2016) (Table 1).

**Table 1:** Overview of the included studies.

**Author/year/ Institution/Country**	**LTx-patients**	**Material**	**Intervention- Type/Time**	**Assessments**	***p*-value, ES**
**Mitchell et al. (****2003****)**, Hospital, Gainesville University Florida, USA.	LTX-candidates (COPD, PF, EMPH, A1AD, BO) waiting list (*n* = 16; 3 women, 13 men), 8 in each group. Age between 49 and 55 years. All LTX patients received Triple-drug immune-suppressive therapy with cyclosporine, prednisone, and azathioprine.	MedX clinical lumbar extension machine; to improve bone mineral density (BMD) in TPL patients with Osteoporosis.	2 Months after LTX start of 6-month resistive strength exercise program. Frequency: 1x/wk, 1 series, 15 up to 20 repetitions through 72 degrees ROM. Non-exercising control group.	BMD lumbar spine, Lumbar extensor strength, AE: Lung Rejection.	CG: Sign. decrease of BMD from 2 up to 8 Months after LTX) below BMD baseline.IT: Sign. improvement of BMD returning to BMD baseline values (ES = 0.52, *p* < 0.05). Extensor Muscle Strength improved in both the CG and the IT, howeverat 36, 48, 60, and 72 degrees ROM, CG improved more than EG with ES varying between −0.04 and 0.27.
**Braith et al. (****2007****)**, Hospital (Dept. applied Physiology), Pilot Study, Gainesville University Florida, USA.	LTX-candidates (COPD, PF, EMPH, A1AD, BO & PH) waiting list, *n* = 30 (11 women, 19 men), 10 in Aledronate-group, 10 in Alendronate&Training-group, 10 in CG. Age between 52 and 56 years.	MedX clinical lumbar extension machine; to improve bone mineral density (BMD) in combination with alendronate or single Alodronate intervention in TPL patients with Osteoporosis.	2 Months after LTX start of 6-month resistive strength exercise program. Frequency: 1x/wk, 1 series, 15 up to 20 repetitions through 72 degrees ROM. The patients in the single Alodronate Group& CG did not participate in a training program.	BMD lumbar spine, Lumbar extensor strength, AE: Lung Rejection.	BMD: Sign. decrease of BMD from 2 up to 8 Months in CG after LTX below BMD baseline. No significant decrease in the single Alodronate group from baseline to 2 months. BMD improved sign. in combined Alendronate & EG (ES = 1.09, *p* = 0.05). Extensor Muscle Strength improved sign. for all ROM in the combined group compared to the CG and single Alodronate group (p < 0.05).
**Ihle et al. (****2011****)**, Schön Klinik Berchtesgadener Land & Klinikum Grosshadern, Ludwig Maximillians Universität Munich, Germany	60 patients 5 years. after transplant, (34 women, 16 men), 30 patients in EG, 30 in CT. Age between 49 and 50.	Exercise training: endurance training; upper and lower limb strength training; Stretches major muscle groups: incl. calf, biceps, hamstrings, quadriceps; range-of-motion exercises of the neck, shoulder and trunk & education program.	Inpatient 5 h of supervised training; 30 min. breathing exercises 30 min. group-aerobic 5x/wk.; education. Pat received endurance, strength; stretching training vs. outpatient CT received standard-physiotherapy.	Cardio-pulmonic exercise testing, 6MWT, SF-36, SGRQ, HRQOL.	Endurance and HRQOl improved in both groups, but there were no sign. group differences for cardio-pulmonary exercise testing nor 6 MWT or any other outcome.
**Langer et al. (****2012****)**, University Hospital, KU Leuven, Belgium.	34 patients after uncomplicated LTX (≤ 6 wks. in hospital (18 woman, 16 men), 18 in IT (9 women, 9 men) & 16 in CT (9 woman, 7 men) age 59 (SD 4) years.	Stationary bicycle & leg press.	12 wk. physical exercise training intervention 3x/wk. initially at 60% for cycling training and 75% for treadmill training. Cycling, walking, climbing stairs, und strength training during 90 min per training. CT received no exercise.	Walking time & intensity, steps/day, 6MWT, Quadriceps& grip strength, inspiratory muscle strength, HRQoL, SF-36.	Sig. difference between EG and CG immediately after the exercise intervention were found for walking time (ES = 0.77, *p* = 0.008), walking intensity, (ES = 2.05, *p* = 0.044), daily steps (ES = 0.92, *p* = 0.004), 6MWT (ES = 0.99, *p* = 0.008), and Quadriceps strength (ES = 1.16, *p* = 0.001). After 1 year daily, significant group differences were yielded for walking time (*p* = 0.006), Quadriceps force (*p* = 0.001), 6-min walking distance (*p* = 0.002) and Self-reported physical functioning (*p* = 0.039).
**Gloeckl et al. (****2015****)**, Schön Klinik Berchtesgadener Land, Germany	83 patients 10 (12) wks. post LTX), (41 women, 42 men), 44 WBVT-EG, 39 CG, Age 55 (SD 9)	Stationary bicycle, Vibrationplatform (Galileo, Novotec Medical GmbH, Pforzheim, Germany	4 wk. in-hospital exercise program. 5-6x/wk.Included medical care, exercise training, breathing therapy, special LTX education, nutritional counseling and psychological support. Endurance training (15 min.) at 60% of peak workrate, strength training (4–5 exercisers for majormuscle groups, 3 x 20 min). Additional 4 x 2 min. Squattraining with vibrations (WBVT-group) or without vibration (CG).	Primary Outcome: 6MWT Sec. outcome PWR, STST, max. muscle strength, HRQoL (CRQ, HADS)	Improvement in 6MWD was sign. (ES = 0.54, *p* = 0.029) higher in the WBVTgroup (83.5 m, compared with CONgroup. Peakworkrate increased sign. (ES = 0.38, *p* = 0.042) more in the WBVT group to the CG.
**Fuller et al. (****2017a****)**, The Alfred-Hospital, Melbourne, Australia	66 patients after LTX, (33 woman, 33 men, age between 38-64 years randomized in 7 wk. and 14 wk. PE program.	Stationary bicycle, treadmill, resistive strength machines or weights.	3x/ wk. 60 min.: Endurance (30 min.) & strength training. Home exercise in 7-wk. group on a stationary bicycle or walking and strength exercises. The home exercise group received education during the first seven wks.	Primary outcome: 6MWT Secondary outcome: Quadriceps and hamstrings strength, HRQol	6MWD improved in both groups with no sign.difference between groups at any time point. Similarly, at 6 months, there was no difference between groups in quadriceps and hamstring strength or mental or physical health domains of HRQoL.
**Fuller et al. (****2017b****)**, The Alfred-Hospital, Melbourne, Australia	80 LTX patients, (43 woman, 37 men, age 45–68 years	Supervised lower limb endurance training incl. treadmill walking and cycle ergometry, & lower limb strength training.	LTX patients randomized randomized in: (1) no supervised upper limb program (NULP) or (2) structured, supervised upper limb program (SULP). 12 week training, 3x/wk. 60 min.: Endurance (30 min.) & group strength training.	Primary outcome: Overall bodily pain (VAS) Secondary outcome: Sf-36, Bodily pain, Strength, Quick Dash & MWT Bodily pain site	VAS bodily pain improved in NULP & SULP over all time points:group x time interaction (*p* < 0.001). *Post-hoc* tests SULP less bodily pain compared to NULP *p* < 0.001) with no diff. between groups at other time points (ES 1.13). Sign. Diff. in sf-36 between groups at 12 wks for SULP (0.05). Strength: SULP sign. stronger (peak force) than NULP 0.037).6MWT improved over time, but no sign. group x time interaction. After 12 wks. SULP had less posterior thoracic pain sited on body chart, compared to NULP (*p* = 0.026). No other diff. across body sites for pain. At 6 weeks, SULP sign. less paracetamol intake (dosage) on outcome measurement day. (*p* = 0.03).

*6MWT, 6-Minute-Walking Test; A1AD, Anti alpha1 antitrypsin deficiency; AE, Adverse effects (lung rejection); BMD, Bone mineral density; BO, Bronchiolitis obliterans; CG, Control group; COPD, Chronic Obstructive Pulmonary Disease; CRQ, Chronic Respiratory Questionnaire; CT, Continuous exercise Training; EG, Exercise Group: EMPH, Emphysema; ES, effect size; HADS, Hospital Anxiety and Depression Scale; HRQoL, Health related quality of life; IG, Interventiongroup; IT, Intervall Training; LTX, lung transplantation; NULP, NO supervised exercise; PF, Pulmonary fibrosis; PH, Pulmonary hypertension; PWR, Peak work rate; QoL, Quality of Life; QWB, Quality of well-being scale; ROM, Range of Motion; SF-36, 36-Item Short Form Survey; SGRQ, St. George's Respiratory Questionnaire; Sign., Significant; STST, Sit-to-stand Test; SULP, Supervised upper limb exercise program; US, Ultra Sound; VAS, Visual Analog Scale; WBVT, Whole-body vibration training; wk., week*.

### Description of exercise principles

The assessment and grading of exercise principles (Hoffman, 2002) reporting (Table 2) meant that one point was given for those cases where a clear description of a training principle was identifiable. Absent or unclear descriptions implied giving zero points for the concerned item.

**Table 2:** Description of reporting of the principles of exercise training.

	**Specificity**	**Progression**	**Overload**	**Initial values**	**Reversibility**	**Dim. returns**	**Total**
Mitchell et al., 2003	1	1	1	1	0	0	4
Braith et al., 2007	1	1	1	1	0	0	4
Ihle et al., 2011	1	0	0	1	0	0	2
Langer et al., 2012	1	1	1	1	0	0	4
Gloeckl et al., 2015	1	1	1	1	0	0	4
Fuller et al., 2017a	1	1	1	1	0	0	4
Fuller et al., 2017b	1	1	1	1	1	0	5
Total	7	6	6	7	1	0	

*Clear description of training principle = 1; Unclear or no description of training principle = 0*.

### Description of the FITT exercise program components

Similar as in commensurable reviews, where FITT components for cardiovascular and resistive exercise were summarized (Knols et al., 2016), characteristics of exercise sessions, episodes, or bouts per week (Hoffman, 2002) were collected. This included descriptions of recovery time following training, both for exercise and control groups where applicable (Bishop et al., 2008). Intensity refers to the amount of work performed or to the magnitude of effort required performing an activity or exercise. Time, length, or duration in which an activity or exercise is performed was captured; e.g., expressed in minutes, together with information about the exercise type (Hoffman, 2002; Ammann et al., 2014; Baschung Pfister et al., 2015). Registering of FITT component and patients' adherence to the intervention was done with two rating categories by two individuals (NF & DK); “reported” = 1 point, “not or unclear/inconsistently reported” = 0 points (Table 3). FITT components were assumed to be mentioned on two occasions within a manuscript; once while the planned training program was detailed, and the second time in the Results section of a manuscript to cover aspects of adherence to the training plan (Table 3).

**Table 3:** Reporting of planned FITT (gray) and adherence FITT components (white).

**Component**	**Frequency**	**Intensity**	**Time**	**Type of exercise**	**Total planned**	**Frequency**	**Intensity**	**Time**	**Type**	**Total Adherence**
Mitchell et al., 2003	0	1	0	1	2	0	0	0	0	0
Braith et al., 2007	0	1	0	1	2	0	0	0	0	0
Ihle et al., 2011	0	0	0	1	1	0	0	0	0	0
Langer et al., 2012	0	1	1	1	3	0	0	0	0	0
Gloeckl et al., 2015	0	0	0	1	1	0	0	0	0	0
Fuller et al., 2017a	0	0	0	1	1	0	0	0	0	0
Fuller et al., 2017b	0	1	0	1	2	0	0	0	0	0
Total	0	4	1	7	–	0	0	0	0	0

*1, FITT component reported; 0, FITT component not reported or unclear/inconsistently reported*.

*FITT, Frequency, Intensity, Time Type; RCT, Randomized controlled trial*.

### Description of the intervention details

A purpose developed guide and checklist was exerted to monitor intervention details (Hoffmann et al., 2014). For each single item on the checklist three rating categories were used. Rating was performed by NF and DK, independent from each other, as either “reported,” “unclear or inconsistently reported,” or “not reported” (Table 4).

**Table 4:** Description of the intervention details (TIDieR- Checklist).

**Item number**	**Mitchell et al. (2003)**	**Braith et al. (2007)**	**Ihle et al. (2011)**	**Langer et al. (2012)**	**Gloeckl et al. (2015)**	**Fuller et al. (2017a)**	**Fuller et al. (2017b)**	**Total per item**
1	+	+	+	+	+	+	+	7
2	+	+	+	+	+	+	+	7
3	+	+	–	?	?	?	?	2
4	?	?	?	+	+	+	?	3
5	+	–	–	–	–	?	+	2
6	?	?	?	?	?	?	?	0
7	-	?	?	?	?	+	+	2
8	?	?	?	?	?	?	?	0
9	+	?	–	?	?	?	?	1
10	–	–	–	–	–	–	–	0
11	–	–	–	–	?	?	?	0
12	–	–	–	–	?	?	–	0
Total per Study	5	3	2	3	3	4	4	

*+, Item reported; ?, unclear or inconsistently reported; –, Item not reported*.

### Description and rating of study quality

A purpose developed checklist for study quality determination (Downs and Black, 1998) was applied by NF and AM to the critique of studies included in this review. Both performed the ratings independent from each other. “The scoring of the last item (“study power”) was modified from a 0–5 scale to a 0–1 scale, where 1 was scored when the authors reported whether and how they determined their sample size a priori (Schoene et al., 2014). One was scored if a power calculation or sample size calculation was present; zero was scored if there was no power calculation, sample size calculation, or justification whether the number of subjects in the study at hand was appropriate. Items 4 (“description of the intervention”) and 19 (“compliance with the intervention”) were scored “yes” if all FITT components were described in the methods and results for the intervention and the control group (if active at all), respectively. According to other recommendations (Kamper et al., 2015; Moseley et al., 2015), we scored item 26 (“losses to follow-up”) “yes” if the dropout rate was less than 15 percent or if an intention-to-treat analysis was conducted. The score from this modified version ranged from 0 to 28, with a higher score indicating higher methodological quality”. (Knols et al., 2016) (Table 5).

**Table 5:** Downs and black methodological quality list.

**Item**																														
	**1**	**2**	**3**	**4**	**5**	**6**	**7**	**8**	**9**	**10**	**11**	**12**	**13**	**14**	**15**	**16**	**17**	**18**	**19**	**20**	**21**	**22**	**23**	**24**	**25**	**26**	**27**	**T**	**P**	**%**
Mitchell et al., 2003	1	1	0	0	1	1	1	0	0	0	0	0	0	0	0	1	1	1	0	1	1	0	1	0	0	0	0	11	28	39.3
Braith et al., 2007	1	1	0	0	1	1	1	1	0	0	0	0	0	0	0	0	1	1	0	1	0	0	1	0	0	0	0	10	28	35.7
Ihle et al., 2011	1	1	1	0	1	1	1	0	1	1	1	0	0	0	0	1	1	1	0	1	1	1	1	0	0	1	0	17	28	60.7
Langer et al., 2012	1	1	1	0	1	1	1	0	0	1	1	0	1	0	1	1	1	1	0	1	1	1	1	1	0	0	1	19	28	67.9
Gloeckl et al., 2015	1	1	1	0	1	1	1	1	0	1	1	0	0	0	1	1	1	1	0	1	0	1	1	1	0	0	1	18	28	64.3
Fuller et al., 2017a	1	1	1	0	1	1	1	1	1	1	1	0	1	0	1	1	1	1	0	1	1	0	1	1	1	1	1	22	28	78.6
Fuller et al., 2017b	1	1	1	0	1	1	1	1	1	1	1	0	1	0	1	1	1	1	1	1	1	0	1	1	1	1	0	22	28	78.6
Total	7	7	5	0	7	7	7	4	3	5	5	0	3	0	4	6	7	7	1	7	5	3	7	4	2	3	3	

*T, Scored points; P, Maximum points, %, Percentage*.

### Data analyses

Benchmarks of Landis and Koch (1977) were used for the calculation and interpretation of between rater agreements (percentage agreement & Cohen's kappa, respectively). The categories were poor (0), slight (0.1–0.20), fair (0.21–0.40), moderate (0.41–0.60), substantial (0.61–0.80), and almost perfect (0.81–1.0) agreement (Landis and Koch, 1977). The reporting items of this systematic review follow the PRISMA statement (Liberati et al., 2009; Moher et al., 2009; Knols et al., 2016). Cohens d effect sizes (ES) were calculated immediately after the end of the PE program in case appropriate (Field, 2014).

To enhance rating consistency two meetings were organized to gear different raters to using the same approach and extract exercise principles, FITT components and adherence information, together with a standardized description of intervention items and methodological quality. In case of disagreement, RHK served as a referee in the familiarization session.

## Results

### Study selection and characteristics

**Figure 1:**
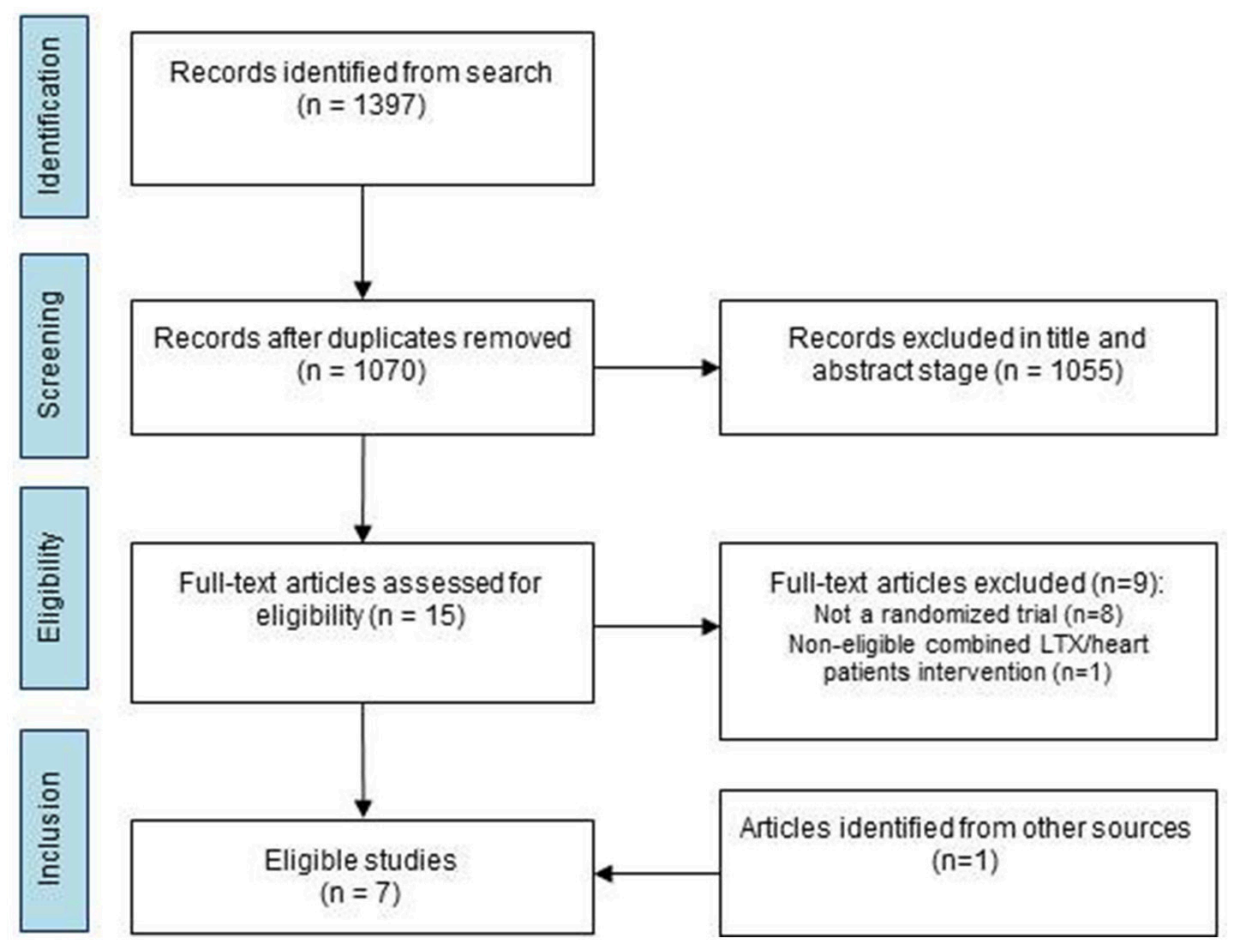
Flowchart of the systematic review. RCT, randomized controlled trial; LTX Lungtransplantation.

The systematic search up to November 2016 provided 1397 citations. Following deduplication, 1070 hits remained. From these, 1055 titles failed meeting the inclusion criteria and were, thus, excluded. Full texts of 15 manuscripts were retrieved and screened. Six studies were at the end available for inclusion. Figure 1 presents the search summary and shows one study could be added following hand searching. The included studies were published from 2003 to 2017. Sample sizes varied between 16 and 80 patients. All studies included adult patients with their age ranging between 45 and 68 years. Altogether, 369 patients were included in the selected articles, 183 females and 186 males. Two studies were performed in Northern America (Mitchell et al., 2003; Braith et al., 2007), three in Europe (Ihle et al., 2011; Langer et al., 2012; Gloeckl et al., 2015) and two in Australia (Fuller et al., 2017a,b). The duration of the PE program in the studies varied between 1 (Gloeckl et al., 2015) and 6 months (Mitchell et al., 2003; Braith et al., 2007; Fuller et al., 2017b). One study of Fuller and colleagues evaluated the effect of a shorter (7 wks.) vs. a longer period (14 wks.) of PE (Fuller et al., 2017a) and another the effect of a supervised vs. a non-supervised exercise group (Fuller et al., 2017b). Two studies reported the effect of resistive strength exercise (Mitchell et al., 2003; Braith et al., 2007), one study the effect of endurance exercise (Langer et al., 2012) and four studies the effects of combined forms of PE (Ihle et al., 2011; Gloeckl et al., 2015; Fuller et al., 2017a,b).

Significant between-groups differences were reported in 5 RCTs for preservation of lumbar bone mineral density (BMD), quadriceps strength, walking time, walking distance, peak work rate, bodily pain VAS /Sf-36, and less pain medication intake (paracetamol) (Mitchell et al., 2003; Braith et al., 2007; Langer et al., 2012; Gloeckl et al., 2015; Fuller et al., 2017b). Conversely, three studies did not report significant between group differences for the primary outcomes 6 MWT, quadriceps and hamstrings strength, exercise capacity and self-reported quality of life (Ihle et al., 2011; Fuller et al., 2017a,b) (Table 1). Effect sizes (Cohens d) (Mitchell et al., 2003; Braith et al., 2007) for significant *p*-values were calculated directly after PE for the outcomes BMD of the lumbar Vertebrae L2-L3 (*r* = 0.52) (Mitchell et al., 2003), and for BMD as a combination of alendronate &PE training (*r* = 1.09) (Braith et al., 2007). Effect sizes for absolute isometric lumbar extensor strength values after PE training at 0 degrees were (*r* = 0.27), 12 degrees (*r* = 0.07), 24 (*r* = 0.18), 36 (*r* = −0.10), 48 (*r* = −0.08), 60 (*r* = −0.21), and 72 (*r* = −0.04) degrees (Mitchell et al., 2003). ES for walking (min /day), movement intensity, daily steps, quadriceps force and 6 Minute Walking Distance (6 MWD) were 0.77, 2.05, 0.92, 1.16, and 0.99, respectively (Langer et al., 2012). The ES for 6 MWD in the study of Gloeckl et al. (2015) was 0.54 and 0.38 for peak work rate (Table 1). Finally, the ES for decreased bodily pain after supervised training was 1.13.

### Synthesized findings exercise principles

The Exercise Principles “specificity” and “initial values” were identifiable and reported in all seven studies (Mitchell et al., 2003; Braith et al., 2007; Ihle et al., 2011; Langer et al., 2012; Gloeckl et al., 2015; Fuller et al., 2017a,b), six of seven studies reported the “progression” and “overload” (Mitchell et al., 2003; Braith et al., 2007; Langer et al., 2012; Gloeckl et al., 2015; Fuller et al., 2017a,b), and the principle “reversibility” was reported only once (Fuller et al., 2017b). “Diminishing returns” was not identified in any study (Table 2).

Reporting of FITT components is recapitulated in Table 3. The mean/median of the mentioning of these components was 2 (range 0–7). “Frequency” was described in none of the studies. Four studies reported training intensity (Mitchell et al., 2003; Braith et al., 2007; Langer et al., 2012; Fuller et al., 2017b). One study reported “time” (Langer et al., 2012), and all seven studies reported “type” of exercise (Mitchell et al., 2003; Braith et al., 2007; Ihle et al., 2011; Langer et al., 2012; Gloeckl et al., 2015; Fuller et al., 2017a,b). All RCTs described 3 or less components. Whether the planned training was feasible for the patient populations; e.g., by detailing aspects of adherence to training based on the FITT components, was reported in none of the seven studies (Table 3). Agreement between raters for these two rating categories (expressed through kappa values) was 0.5 (95%CI 0.3–0.7).

Table 4 expresses the results following assessment of the studies with the purpose developed guide and checklist for monitoring intervention details (Hoffmann et al., 2014). Two to five (median three) items were mentioned in the studies, ranging between 0 and 7 items mentioned for the individual studies (median 1.5). The interrater agreement (kappa) was 0.7 (95%CI 0.5–0.8).

### Methodological quality

Methodological flaws in relation to the transparency of reporting of the PE interventions were failure of detailed reporting of the exercise program (item 4), the blinding of the treatment groups (item 14), the proportion of those wo agreed in the trials (item 12), and adjustment of adherence in the exercise program (item 19). The overall methodological quality, expressed as a percentage (Downs and Black, 1998) in Table 5, was 60.7% (median 64.3%) and resulted in an “moderate to substantial” inter-rater agreement (kappa) of 0.7 (95% CI 0.6–0.8).

## Discussion

The aim of this systematic review was to evaluate the replicability of exercise interventions for LTX patients by assessing the details of the description of these interventions in randomized control studies. The results showed that PE training components were not described with a level of detail that would guarantee full replicability for researchers wanting to replicate the intervention or for clinicians wanting to implement successful interventions for their patients. Based on the partially sparse descriptions clinicians cannot be confident in replicating the exercise programs such that similar effects are attained for their patients. There was a lack of detail regarding the reporting of items that would be essential for good replicability; e.g., giving sufficient information about the frequency of training, the intensity needed to receive the results, the time needed for one training and the exercise type that has to be applied (Knols et al., 2016).

The identification of these vestigial descriptions of intervention plans that prevent program replication with confidence for both researchers and practitioners is an important uncovered research gap. Although all seven trials reported which type of training was used, none of the studies provided reports on how well patients were able to comply with their prescribed training. Similar results were observed for the description of other important aspects of training components. This is a point of concern because a lack of detailed description regarding both the planning and the adherence to planning of training will make it difficult identifying the true value of an intervention in a clinical setting (Reimer, 1998).

LTX-patients may attain significant improvement in pulmonary function and exercise capacity; however, peak exercise performance remains often suboptimal. Previous studies that evaluated cardio-pulmonary performance following single- and double-LTX report a reduced anaerobic threshold and reduced maximum oxygen consumption even without apparent significant cardiac or ventilatory limitations in the patients (Williams et al., 1992; Evans et al., 1997; Schwaiblmair et al., 1999). LTX- candidates with advanced lung disease (e.g., COPD), are often severely deconditioned and have reduced skeletal muscle mass and weakness (1999, 1999; Ahya and Kawut, 2005). Weak LTX patients are less likely to favorably respond to PE interventions (Vivodtzev et al., 2011). Seen from this perspective it becomes clear why it is so important to be able and identify replicable successful interventions. The proper description and application of interventions with sufficient details will not only prevent wasteful research from happening but will also potentially increase the impact of research on the health of patients.

In this review *specificity, initial values, overload* and, *progression* were the most frequently applied (i.e., explicitly reported) training principles, in 7/7 (specificity & initial values) and 6/7 (overload & progression) of the reviewed studies respectively. In accordance with this reporting, most exercise trials clearly outlined training progression and reported their intervention to be specifically designed to the target population. These aspects warrant reproducibility with respect to these training principles. In contrast to this, however, *reversibility values* (1/7) and *diminishing return* (0/7) were only once or not at all considered. This is a point of concern because without knowing the baseline fitness levels of studied participants, it is difficult to generalize the findings to a clinical setting. In clinical settings important reductions in skeletal muscle force immediately after lung transplantation have been mentioned with striking differences in recovery behavior observable between men and women (Maury et al., 2008). Delayed recovery of exercise capacity is, furthermore, secondary to slow recovery of muscle strength in these patients (Walsh et al., 2013). It seems fair to speculate that the mechanisms behind slower recovery rates for women might be better elucidated when information about initial fitness levels would be available and could be compared to sex specific reference values.

Moreover, interpretation of exercise results is hindered due to a lack of reporting exercise frequency (0/7), intensity (4/7) and, Time (1/7). Information about these FITT components of exercise are, however, important since appropriate training parameters in terms of time, frequency, and intensity seem necessary to achieve improvements in limb muscle function and exercise capacity of LTX patients (Langer, 2015).

Current guidelines for pulmonary rehabilitation do not include lung transplant candidates or recipients (Spruit et al., 2013) indicating the existing unclarity related to whether LTX patients can reap benefits from specific rehabilitation or exercise programs. This prompted a multi-disciplinary group of experts in solid organ transplantation; e.g., clinicians, researchers, administrators and patient representatives, to formulate research recommendations in this area (Mathur et al., 2014). The number three place in the top research priorities identified by the group was given to knowledge translation about current evidence together with identified gaps in evidence to relevant stakeholders (Mathur et al., 2014). This is expected to give a boost in addressing future research in exercise for solid organ transplant. Based on the findings of our review the reporting of exercise intervention programs that would allow full replication of the interventions should be added to such an agenda. The reporting of future RCTs evaluating PE interventions in LTX patients may ameliorate when standards of trial reporting (Moher et al., 2001) are already taken into account in the trial planning phase, together with checklists helping in the detailed description of interventions.

### Study limitations

There were some limitations related to this systematic review that should be mentioned. To the best of our knowledge, this systematic review is the first to investigate the replicability of exercise interventions in LTX patients by assessing reporting of exercise training principles. This makes that instead of focusing on the actual intervention outcomes the review rather directs attention on the reporting of intervention content. In our endeavor to achieve a robust systematic review, we developed and documented the methods (e.g., a systematic search strategy and several worksheets for collecting and synthesizing the data) in advance. Due to the number of existing trials on LTX exercise interventions, we decided to focus exclusively on RCTs to ensure high external validity. However, some limitations are a consequence of this approach. Because we restricted our search to English language publications we might have missed out on studies reported in other languages. This gives a possibility that important RCTs published in other languages were missed. Second, because of the scope of the review, we did not perform meta-analyses of RCT results. Therefore, we cannot make any recommendations concerning preferable exercise interventions for LTX patients. However, due to the fact that no guidelines for this patients group currently exists (Spruit et al., 2013) we estimate this limitation not being grave. Currently we cannot refer to credible literature for training recommendations based on best available evidence for LTX patients. Furthermore, a selection bias may have been present, as the database search was performed by a professional librarian up to November 2016, hereafter the databases were checked monthly by RHK and NF up to December 2017.

## Conclusions

This review showed that replicability of many exercise interventions in LTX is not warranted due to poor descriptions of important items related to training. In particular there were insufficiently detailed reporting of training principles and FITT components in programs developed for LTX. When training program details are lacking or insufficiently described, translation into clinical practice is left with uncertainties in relation to the effectiveness of the programs. Future interventions that aim to train LTX patients should spent effort in writing reports in which the intervention is detailed to such an extent that full replicability in clinical settings can be guaranteed.

## Author contributions

RK conceived the methodology and carried out quality assessment, data analysis, and manuscript writing. NF participated in methodology conception, data assessment, and analyses and manuscript writing. DK and AM carried out data collection and analysis, and manuscript writing. EdB supervised progress, helped with methodology conception, manuscript writing & critical revision for scientific content. All authors read and approved the final manuscript.

### Conflict of interest statement

The authors declare that the research was conducted in the absence of any commercial or financial relationships that could be construed as a potential conflict of interest. The reviewer RA and handling Editor declared their shared affiliation at time of review.

## Abbreviations

BMD: bone mineral density
COPD, Chronic obstructive pulmonary disease, FEV1: Forced expiratory volume in 1 second
FITT, Frequency, Intensity, Time: Type
HIIT: High intensity interval training
LTX: Lung transplantation
PE: Physical exercise
VO2max, Maximum oxygen consumption, VO2peak: Peak power output.
